# The Utilization of Dehydroepiandrosterone as a Sexual Hormone Precursor in Premenopausal and Postmenopausal Women: An Overview

**DOI:** 10.3390/ph15010046

**Published:** 2021-12-29

**Authors:** Justine Tang, Li-Ru Chen, Kuo-Hu Chen

**Affiliations:** 1 School of Medicine, College of Medicine, Taipei Medical University, Taipei 110, Taiwan; justinetang627@gmail.com; 2 Department of Physical Medicine and Rehabilitation, Mackay Memorial Hospital, Taipei 104, Taiwan; gracealex168@gmail.com; 3 Department of Mechanical Engineering, National Yang Ming Chiao Tung University, Hsinchu 300, Taiwan; 4 Department of Obstetrics and Gynecology, Taipei Tzu-Chi Hospital, The Buddhist Tzu-Chi Medical Foundation, Taipei 231, Taiwan; 5 School of Medicine, Tzu-Chi University, Hualien 970, Taiwan

**Keywords:** dehydroepiandrosterone, DHEA, DHEAS, menopause

## Abstract

Dehydroepiandrosterone (DHEA), and its metabolite, dehydroepiandrosterone sulfate ester (DHEAS), are the most abundant circulating steroid hormones, and are synthesized in the zona reticularis of the adrenal cortex, in the gonads, and in the brain. The precise physiological role of DHEA and DHEAS is not yet fully understood, but these steroid hormones can act as androgens, estrogens, and neurosteroids, and perform many roles in the human body. Since both levels decline with age, use of DHEA supplements have gained more attention due to being advertised as an antidote to aging in postmenopausal women, who may have concerns on age-related diseases and overall well-being. However, current research has not reached an overall consensus on the effects of DHEA on postmenopausal women. This overview is a summary of the current literature, addressing the metabolic pathway for DHEA synthesis and utilization, as well as the effects of DHEA on premenopausal and postmenopausal women with disease states and other factors. As for the therapeutic effects on menopausal syndrome and other age-related diseases, several studies have found that DHEA supplementations can alleviate vasomotor symptoms, preserve the integrity of the immune system, reduce bone loss, and increase muscle mass. Intravaginal DHEA has shown significant beneficial effects in menopausal women with severe vulvovaginal symptoms. On the other hand, DHEA supplements have not shown definitive effects in cardiovascular disease, adrenal insufficiency, insulin sensitivity, and cognition. Due to inadequate sample sizes and treatment durations of current studies, it is difficult to assess the safety and efficacy of DHEA and draw reliable conclusions for the physiological role, the optimal dosage, and the effects on premenopausal and postmenopausal women; therefore, the study of DHEA warrants future investigation. Further research into the roles of these steroid hormones may bring us closer to a therapeutic option in the future.

## 1. Introduction

Dehydroepiandrosterone (DHEA), an endogenous 19-carbon steroid hormone, and its metabolite, dehydroepiandrosterone sulfate ester (DHEAS), are the most abundant circulating steroid hormones, and are predominantly secreted by the zona reticularis of the adrenal cortex, the gonads, as well as the brain. Due to the long plasma half-life, most DHEA becomes DHEAS in the plasma; therefore, DHEAS is present in a much greater concentration compared to DHEA [1], and is reserved and converted into specific hormones when needed. Generally, both levels decline in concentration with age [2], and age-related diseases become more prevalent.

In the fetus, DHEA levels are high, but drop shortly after birth until adrenarche. Adrenal cortex starts producing these hormones during puberty, which peaks in early adulthood. At around 25 years of age, plasma DHEAS declines gradually thereafter at a rate of 10% per decade [2,3]. Throughout the entire adult lifespan, normal DHEAS values range from 0.3 to 3.5 µg/mL. Women between the ages of 35–45 and 45–55 have normal ranges of 1.0–3.0 µg/mL and 0.7–1.6 µg/mL, respectively [4]. By 75 years of age, the plasma DHEAS levels are about 80% lower than those at 25 years of age due to decreased adrenal production [1].

Nowadays, both DHEA and DHEAS play significant roles in protecting against high cortisol levels and in synthesizing androgens and estrogens; however, their physiological effects and mechanisms of action are not fully understood. Biological functions of DHEA include acting as an androgen, as an estrogen, and as a neurosteroid. DHEA and DHEAS are able to perform many roles in the human body. Biological actions include neuroprotection, neurite growth, and antagonistic effects on oxidants and glucocorticoids. In addition, metabolic, immune-modulating, and anticancer effects have been attributed to these steroids [5]. There are many beneficial effects of DHEA, for instance improving mood state and correcting hormone levels. With optimal concentrations of DHEA, these hormones can improve psychological well-being, extend lifespan, protect brain function and development, and reduce age-related neurodegenerative disorders.

Both DHEA and DHEAS concentrations steadily decrease with age and this reality has led to the suspicion that development of DHEAS deficiency plays a role in the decline of metabolic and physical function, resulting in the development of age-related diseases due to immune system dysfunction, activation, and oxidative stress [6,7]. DHEA has been named the “fountain of youth” or an antidote to aging because of its peculiar characteristics and its positive correlation between high DHEA levels and good health. Due to the favorable effects on anti-aging, immune system, neurobehavioral functions, and well-being, there has been a growing interest in DHEA replacement therapy, especially within the elderly population. DHEA is easily acquired in the United States since it is sold over the counter and remains as a food rather than a drug subject to the Food and Drug Administration regulation. In other countries, DHEA is formally regulated as a drug, but is freely available at health food shops or on the internet [8]. The fact that DHEA is widely sold as supplements may hinder possible scientific evaluation of its potential. Moreover, the effectiveness of DHEA is controversial and its success rates vary.

## 2. Materials and Methods

The current review was modeled according to the Preferred Reporting Items for Systematic Reviews and Meta-Analyses (PRISMA) statement (registration number: 10.17605/OSF.IO/SJNXZ at the website: https://osf.io/search/?q=SJNXZ&page=1, accessed on 22 December 2021). In the current review, all the reference articles were retrieved from the database Ovid Medline by using the search terms “dehydroepiandrosterone”, “menopause”, “DHEA”, and “DHEAS” for the topic. Only full-text articles were considered for further screening and inclusion. Using the database Ovid Medline up to 31 July 2021, we conducted a literature search as to identify potential articles in the database. Research before 1980 and duplication articles were excluded during the screening process. After initial evaluation, two experts in the field independently inspected and evaluated potentially eligible studies for exclusion and inclusion. Articles with poor study design or those with unmatched outcomes were deemed ineligible for the study. During the period of paper selection, disagreements between the two reviewers were resolved by mutual discussion until a consensus was reached.

**Figure 1:**
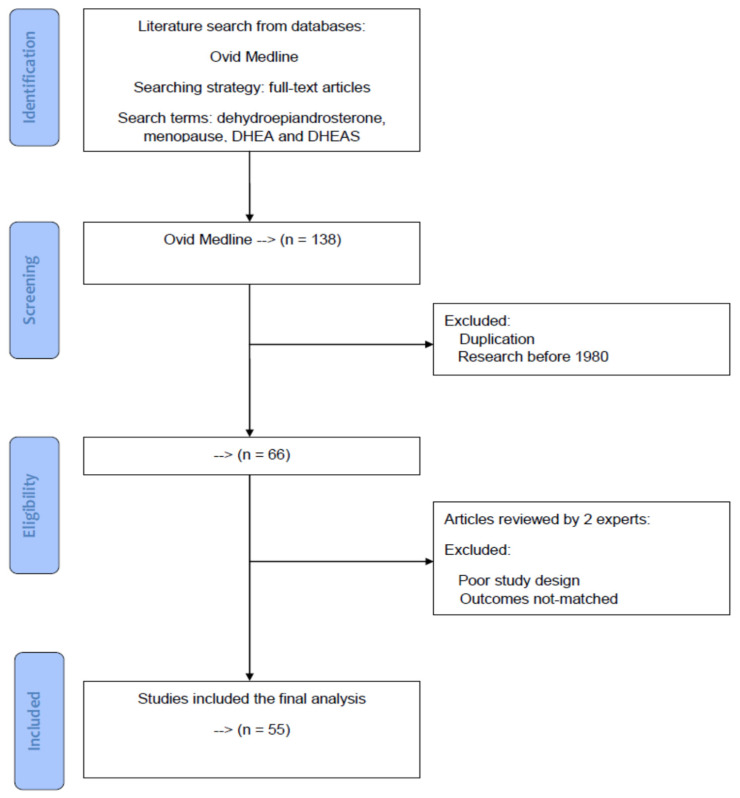
A diagram of article selection to illustrate the processes of database identification, article screening, confirmation of eligibility, and final inclusion based on the PRISMA statement.

Based on the PRISMA statement, a diagram of paper selection is shown in Figure 1 to illustrate the processes of database identification, paper screening, confirmation of eligibility and final inclusion. Using the aforementioned specific search terms and strategy, there were 138 Medline articles on the topic. During the screening process, duplicated articles and research before 1980 were excluded. After excluding, 66 articles were considered for further analysis. By examining the sources based on year, authors, research designs, and clinical outcomes, two reviewers independently inspected and evaluated each article for potential eligible studies. Disagreements between the two reviewers were reassessed, discussed, and resolved until a mutual agreement was reached. Articles with unmatched outcomes or those with poor study design were also excluded, and finally 55 eligible articles for the topic were enrolled in this review.

## 3. DHEA: Mechanisms of Actions

Various studies have been conducted to investigate the effects of DHEA on diseases, such as cancer, diabetes, obesity, depression, sexual dysfunction, aging, and osteoporosis [9]; however, the mechanisms and nuclear steroid receptors of DHEA and DHEAS are not fully understood. The mechanism behind DHEA is complex since its actions vary according to the androgenic or estrogenic effects, gender, and age. Both DHEA and DHEAS are produced mainly from the zona reticularis of the adrenal cortex via classical biosynthetic pathway of pregnenolone, which is derived from plasma low-density lipoprotein and 17α-hydroxypregnenolone (17α-OHPreg), while the rest are produced from the brain and ovarian theca cells in women in response to adrenocorticotropic hormone (ACTH) [10]. In females, the effects of DHEA may vary whether she is premenopausal or postmenopausal. When DHEA is converted to DHEAS in the liver, it acts as a universal precursor for androgens and estrogens. A number of these androgens and estrogens are synthesized in the peripheral tissues by enzymes, which enables them to transform from DHEA to sex hormones including testosterone, dihydrotestosterone, androstenedione, and estrogens [1].

**Figure 2:**
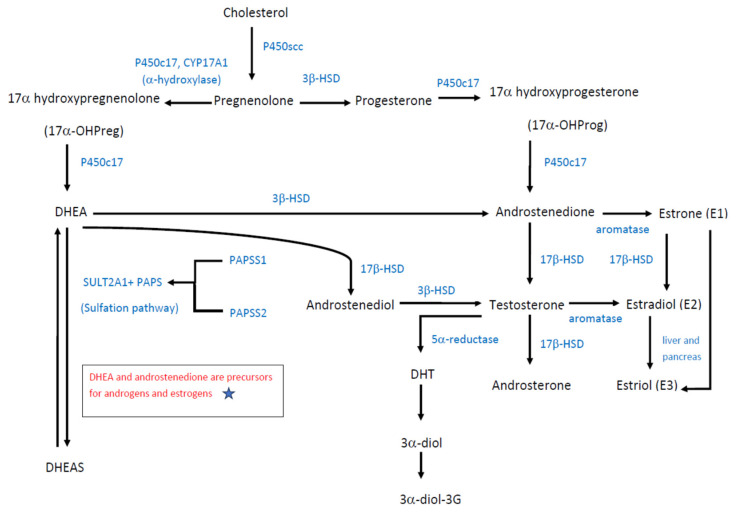
Biosynthesis of DHEA.

Figure 2 illustrates the biosynthesis of DHEA, its precursors and metabolites. Similar to other neurosteroids, DHEA is derived from cholesterol and converted to pregnenolone by side chain cleavage enzyme, P450scc. The conversion of pregnenolone to DHEA produces an intermediate 17α-OHPreg, which is formed by the catalyzation process of an α-hydroxylase, cytochrome P450 17A1 (P450c17, CYP17A1). DHEA can be further converted to DHEAS by DHEA sulfotransferase (SULT2A1) [3]. SULT2A1 is highly expressed in the adrenal cortex, and it catalyzes the conversion of DHEA to DHEAS, which has a longer half-life and higher clearance compared to DHEA [11,12,13]. In addition, cytochrome P450c17 serves as an enzyme controlling the biosynthesis of glucocorticoids and sex steroids and has a dual enzymatic activity, so it can also catalyze progesterone into 17α-hydroxyprogesterone (17α-OHProg), which is further converted to androstenedione. 17α-OHPreg and 17α-OHProg are precursors of cortisol biosynthesis, while DHEA and androstenedione are precursors for androgens and estrogens [14].

In addition, DHEA is a crucial intermediate in maintaining steroidogenesis. Skeletal muscle is a sex steroid-sensitive tissue and is capable of locally synthesizing 5α-dihydrotestosterone (DHT) from either testosterone or DHEA. Testosterone is directly metabolized to DHT through 5α-reduction, while DHEA is metabolized to DHT with the assistance of 3β-HSD, 17β-HSD, and 5α-reductase enzymes [15,16]. As a potent androgen, DHT can be converted to androstane-3α,17β-diol (3α-diol), and subsequent 3α-diol-3-glucuronide (3α-diol-3G), thus activating the glucose metabolism-related signaling pathway [15] (Figure 2).

Adrenal androgens, compounds of 19-carbon steroids, are mainly synthesized in the zona reticularis of the adrenal cortex. Adrenal androgens include DHEA, DHEA-S, androstenedione, and androstenediol. Androstenedione is an androgenic steroid produced by the testes, adrenal cortex, and ovaries. While androstenedione is converted to testosterone and other androgens, it is also a precursor of estrone (E1). Androstenediol is a direct metabolite of DHEA through the catalyzation of 17β-HSD, and the precursor of sex hormones testosterone and estradiol (E2). Androsterone is an end product produced during the stepwise breakdown of androgens, possessing a much weaker potency than testosterone.

In contrast to testosterone, 5α-reduced androgens, such as DHT and its metabolic compounds, have tissue specific actions. DHT is derived from testosterone but is more potent than testosterone because that it binds more strongly to androgen receptors. It is produced in the skin and reproductive tissue. DHT is rapidly converted to another 5α reduced androgen, androstane-3α, 17β-diol (3α-diol) in many tissues including skin, accessory sex glands, brain, heart muscle and salivary glands. As a metabolite of DHT, the other form of 5α, reduced androgen, 3α-diol glucuronide (3α-diol G) is predominantly synthesized in sexual tissue and skin. The concentration of 3α-diol and 3α-diol G is greatly increased in hirsute women, and can be a good marker of hirsutism and virilization.

Estrogens can be classified into estrone (E1), estradiol (E2), and estriol (E3). Among them, estradiol (E2) is the most potent estrogen during reproductive age, while estrone (E1) is the predominant circulating estrogen after menopause. However, estriol (E3) is produced in a large amount by the placenta and serves as the major estrogen during pregnancy. Catalyzed by the action of aromatase, androstenedione and testosterone can be further converted to estrone (E1) and estradiol (E2), respectively (Figure 2).

As show in Figure 2, steroidogenesis including the synthesis of DHEA, androstenediol, androstenedione, testosterone, and androsterone is largely dependent on the catalyzation of P450c17, 3β-HSD and 17β-HSD. Most reactions of androgen products are irreversible, while the only reversible reactions in androgen biosynthesis are the reduction of androstenedione to testosterone. The equilibrium favors the synthesis of more potent sex steroids testosterone and estradiol, over the weaker 17 ketosteroids, androstenedione, and estrone.

The doses of DHEA given and concentrations are not a proportional relationship. The concentrations of DHEA depend on the distribution and amount of key enzymes including P450c17, 3β-HSD, 17β-HSD, SULT2A1, and PAPS, rather than on the doses of DHEA given. Moreover, the balance of DHEA and its storage form DHEAS is maintained by the combined actions of SULT2A1 and PAPS. Figure 2 also depicted the sulfation pathway of DHEA conversion to DHEAS. All human sulfation pathways require high-energy sulfate donor 3′-phosphoadenosine-5′phosphosulfate (PAPS), which is produced by isoforms PAPSS1 and PAPSS2. If there is a PAPSS2 deficiency, PAPSS1 cannot compensate for the loss of PAPSS2 gene function. PAPSS2 mutations can present with androgen excess and skeletal malformation. Functional PAPSS2 is required for efficient DHEA sulfation, which involves its interaction with SULT2A1 [17]. Once the SULT2A1 level decreases, DHEAS levels also decrease [13]. Sulfation of DHEA reduces the level of non-sulfated DHEA for conversion to androgens; thus, impaired DHEA sulfation produces excess androgen, which can cause disorders like polycystic ovary syndrome [17]. Conversely, DHEAS can then be converted to androstenedione, testosterone, and DHT [18].

To date, it remains unclear when and how much DHEA (or DHEAS) undergoes transformation to androgens and estrogens. What we know is that the transformation is closely related to the amount and distribution of key enzymes responsible for the catalyzation of sexual steroids. It implies that the distribution of key enzymes 3β-HSD and 17β-HSD determines where and how much DHEA is concerted to androstenedione, androstenediol, testosterone, and androsterone. Therefore, the biological variability (form and amount) of steroid hormones is predominantly regulated by the key enzymes (3β-HSD, 17β-HSD, and aromatase) in target organs.

The 3β-hydroxysteroid dehydrogenase (3β-HSD) is an enzyme that catalyzes the biosynthesis of the steroids, including pregnenolone to progesterone, DHEA to androstenedione in the adrenal gland, and androstenediol to testosterone. It is also present in other steroid-producing tissues, including the ovary, testis, and placenta. In fact, 3β-HSD is essential for the biosynthesis of all classes of hormonal steroids, namely glucocorticoids, mineralocorticoids, androgens, and estrogens. In humans, there are two 3β-HSD isozymes encoded by the HSD3B1 and HSD3B2 genes.

The 17β-hydroxysteroid dehydrogenases (17β-HSD) are responsible for the conversion of 17-ketosteroids including DHEA to androstenediol, androstenedione to testosterone, and estrone (E1) to estradiol (E2). It is recognized to catalyze the redox reactions of sex steroids. The distribution of 17β-HSD determines the actions of steroidogenesis and steroid metabolism. Accordingly, serum testosterone levels are controlled by the conversion of high androstenedione levels into testosterone by 17β-HSD3 in the gonads and by 17β-HSD5 in extragonadal tissue and in Leydig cells. 17β-HSD3 activity is the only reversible enzymatic reaction in testosterone biosynthesis; however, its action is insignificant in the adrenal glands. Genes coding for 17β-HSD include HSD17B1-HSD17B 14.

With increasing levels in testosterone and DHEA, both Akt and protein kinase C-ζ/λ phosphorylations are enhanced, which play important roles in glucose transporter-4 (GLUT-4) regulated signaling pathways. GLUT-4 protein expression is induced in skeletal muscles, and both testosterone and DHEA are able to enhance GLUT-4 translocation; however, testosterone responses are more sensitive. In skeletal muscles, locally synthesized testosterone or DHT affects both the expression and translocation of GLUT-4 via Akt and PKC-ζ/λ; therefore, DHT inhibitor is able to block GLUT-4 protein expression and Akt and PKC-ζ/λ phosphorylations. According to one study, enhancement of enzyme activities, hexokinase, and phosphofructokinase is caused by upregulation of GLUT-4, and it will lead to glycolysis in the skeletal muscle [16]. Moreover, DHEA can be converted to androstenedione and testosterone, which can be aromatized to estrogens (estrone and estradiol) (Figure 2). The 5-androstene-3β,17β-diol (5-diol) can bind to both androgen and estrogen receptors [15].

Although the functions of DHEA are mainly via its roles of endogenous precursors to more potent androgens including testosterone and DHT, DHEA, per se, can directly bind to androgen receptors to act as a low affinity, weak partial agonist. Most of the time, it can be viewed as a weak androgen, while sometimes it competes with the full agonist testosterone and behaves as an antagonist, depending on the circulating testosterone and DHT levels.

Besides, DHEA has also been noted to bind to the estrogen receptors ERα and ERβ. It is recognized as a partial agonist of the ERα with a weak efficacy; however, it acts as a full agonist of the ERβ with a maximal response similar to or greater than that of estradiol, suggestive of it stronger actions over the uterus, breast, brain, colons, and lung epithelium, where ERβ is higher expressed.

**Figure 3:**
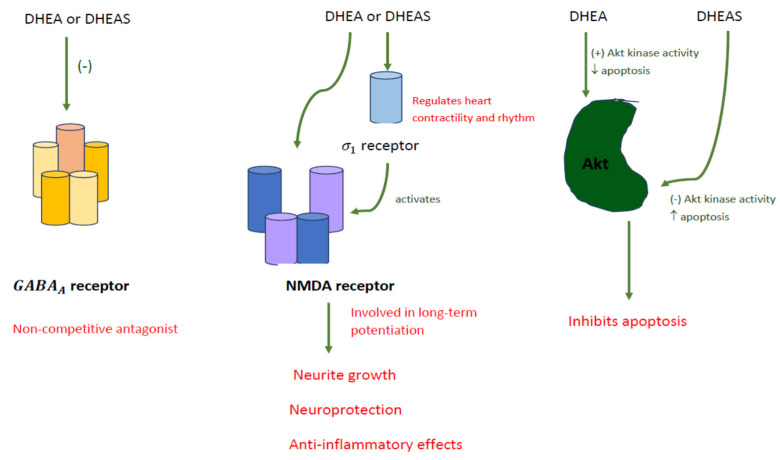
DHEA and DHEAS acting as neurosteroids.

Although these aforementioned hormones are mainly produced by the adrenal cortex and the gonads, they can also be synthesized de novo in the brain, and serve as precursors for androgenic and estrogenic steroids. In the brain, both DHEA and DHEAS can act as neurosteroids, which combine with neurotransmitter receptors involved in learning and memory, such as γ-aminobutyric acid type A (GABAA) receptor, N-methyl-D-aspartate (NMDA) receptor, and the sigma-1 receptor (σ1) [19] (Figure 3). They act as noncompetitive antagonists when combining with the GABAA receptor, but agonists when combining with the σ1 receptor [5,20], which subsequently activates the NMDA receptors [21], a key factor involved in the development of long-term potentiation. By directly stimulating the NMDA receptor, DHEA is able to influence neurite growth and provide neuroprotective and anti-inflammatory effects within the brain [3,21,22]. DHEAS affects sigma receptors, which are thought to be important functional modulators of glutamatergic activity in the hippocampus [5]. The σ1 receptor is expressed in the heart, and it contains a steroid-binding component, which works alongside DHEA. It is believed that the σ1 receptor interacts with ionic channels and calcium influx in the heart, thus regulating the cardiac contractility and rhythm. Likewise, DHEAS has the same negative chronotropic effect on cardiomyocytes, but shows delayed response compared to DHEA [20] (Figure 3).

Actions of DHEA and DHEAS in the brain consist of neuroprotection, neurogenesis and neuronal survival, apoptosis inhibition, production of catecholamine, and anti-glucocorticoid effects. In terms of neuroprotection, DHEA affects cell viability, whereas DHEAS has no effect in SK-N-SH human neuroblastoma cells [23]. In the aspect of neurogenesis and neuronal survival, steroid levels are sensitive to intrinsic factors, such as aging. Aging corresponds with a decrease in neurogenesis in the hippocampus, which can be reversed by lowering corticosterone levels [19,23]. DHEA can increase serotonin levels, increase proliferation of neural stem cells, and manage the neurons being produced. Brain derived neurotrophic factor (BDNF), a mediator of DHEA, differentiates stem cells to neural cells, and plays an important role in neurogenesis. Moreover, BDNF assists in the survival and differentiation of serotonin sensitive tissues, which may be important for the treatment of depression and protection against stress-induced damage [24]. In addition, DHEA increases Akt protein kinase, which acts as an apoptosis inhibitor, whereas DHEAS decreases Akt kinase activity and increases apoptosis [21] (Figure 3). Increased catecholamine synthesis occurs when DHEAS increases TH mRNA and TH protein. Secretion of catecholamines from secretory vesicles occurs when both DHEA and DHEAS stimulate depolymerized actin. Lastly, DHEA backward inhibits the nuclear translocation of glucocorticoid receptors [21].

## 4. The Effects of DHEA on Premenopausal and Postmenopausal Women

Many have proposed both DHEA and DHEAS are important components for estrogen and androgen production, and the treatment with DHEA may ameliorate hormone deficiency symptoms in postmenopausal women. Research investigating the physiologic decline in DHEA and aging, as well as changes in cardiovascular diseases, metabolism, immune systems, bone, cancer, and cognitive effects has brought about insight into the possible effects DHEA may have. DHEA, DHEAS, and androstenedione are precursors for the production of estrogens and androgens. In premenopausal women, the adrenals, ovaries, and peripheral tissues produce approximately 6–8 mg of DHEA per day [15]. With aging process, the use of DHEA in postmenopausal women has been a controversial topic among researchers. Some clinical trials have suggested that DHEA treatment increases sexual function, lipid metabolism and insulin sensitivity, bone density, cognitive performance, and sense of well-being or mood in both healthy elderly and those with illnesses, hence DHEA treatment may yield beneficial effects and reduce both hormone deficiency and postmenopausal symptoms. On the other hand, other studies have shown no therapeutic effects, especially from oral DHEA, and people with intact adrenal function do not necessarily benefit from DHEA supplementation. Currently, there are no definitive conclusions regarding the effects of DHEA on humans.

### 4.1. Vulvovaginal and Endometrial Atrophy

The vagina contains steroidogenic enzymes that are able to transform DHEA into estrogens. Estrogen plays a major role in the vaginal wall, which influences squamous epithelium, lamina propria, and smooth muscle layer. In the lamina propria, estrogen manages vasodilation, while the estradiol (E2) keeps epithelium dense with more mature cells, sustains vaginal smooth muscle, and contributes to tissue elasticity. Estrogen deficiency on the vagina would have adverse effects on sexual function, leading to bleeding and trauma. There would be a decrease in collagen, blood flow, and lubrication, all of which would increase susceptibility to infection [25]. The loss of collagen, elasticity, secretion, and muscular components can cause shortening and narrowing of the vaginal canal [26].

Before a woman reaches menopause, the cyclic secretion of progesterone by the ovaries protects the endometrium. At the time of menopause, the secretion of DHEA decreases to around 60% [27], and the production of estrogen stops, resulting in low E2 levels [26]. The endometrium is free of estrogenic stimulation, thus eliminating utilization of progestin therapy and fear of progestin-induced breast cancer [28]. In other words, after menopause, progesterone is no more needed to protect potential estrogenic stimuli. Due to the absence of enzymes that transform DHEA into estrogens, endometrium is unstimulated, and aromatase is undetectable. About 20% of postmenopausal women are asymptomatic due to sufficient supply of sex steroids from DHEA, but approximately 60% of postmenopausal women have one or more symptoms of menopause [26,28].

Due to the decreased circulating E2 concentration, menopause may be accompanied by symptoms such as vaginal dryness, dyspareunia, dysuria, irritation, and vulvovaginal atrophy [25,26,27]. Vulvovaginal atrophy results in decrease in fluid secretion, decrease in lactobacilli, and increase in vaginal pH. In postmenopausal women, due to changes in the vaginal microflora, the decrease in glycogen is closely related to the increase in pH, so repeated urinary tract infections and vaginal discharge may occur. Likewise, the symptoms of vulvovaginal atrophy can affect daily activities, such as walking, sitting, and exercising, which lead to negative impacts on sexuality, and physical and mental well-being, emotional distress, and quality of life. If left untreated, patients with vulvovaginal atrophy may experience ulcers, petechiae, and tearing [26].

Although estrogen therapy benefits vasomotor symptoms, 40% of women still have vaginal symptoms [25]. According to the Women’s Health Initiative Study, the recommended treatment is using the lowest dose of intravaginal estrogen for the shortest duration. With the administration of 0.50% DHEA, the serum DHEA remains within normal postmenopausal values [25]. Intravaginal DHEA improves vaginal pH, epithelial cell counts, and relieves dyspareunia. Based on the Menopause-Specific Quality of Life (MENQOL) Questionnaire, oral DHEA has shown no improvement in menopausal symptoms, whereas vaginal DHEA has shown improvement in women with vaginal atrophy [29]. Treatments for vulvovaginal atrophy with estrogens and their analogues are used for the epithelial layer, while non-estrogenic treatments can avoid potential stimulatory effects of estrogens. Women who concerned about cancer risks or who have estrogen-sensitive tumors should not use vaginal estrogen [26]. Despite a lack of studies supporting the use and a lack of larger studies evaluating the risks and benefits of vaginal DHEA, DHEA may be helpful in those with severe vulvovaginal symptoms [25].

### 4.2. Cardiovascular Disease

Many studies have shown those who have low DHEA may be prone to cardiovascular diseases; therefore, the correlation between the two has been contended by several researchers. Studies have confirmed that DHEA is inversely associated with cholesterol levels, obesity, and diabetes, which may play an important role in the pathogenesis of coronary artery disease and heart failure. DHEA is a potent inhibitor of fibroblast growth and carcinogenesis in cell culture, which may be a reasonable mechanism between DHEA and coronary diseases [2]. Some studies have reported that DHEA production is inhibited in people with heart failures, which prevents the cardioprotective action [20]. Moreover, DHEA can reverse cardiomyocyte hypertrophy, improve collagen and fibronectin formation and function, and decrease myocardial fibrosis. DHEA treatment also improves the cardiac index and inhibits right ventricular capillary rarefaction, fibrosis, and oxidative stress [30].

Since DHEAS progressively declines with age, many believe the diminution may be related to increasing the risk of cardiovascular diseases. However, results have been obscured due to inconsistent findings. Shufelt et al. evaluated the correlation between lower circulating DHEAS and cardiovascular diseases in the Women’s Ischemia Syndrome Evaluation (WISE) study [31]. This study claimed that DHEAS might play a direct role in the cardiovascular disease mortality risk because of its beneficial effects on hormones, inhibition of platelet aggregation, sensitivity to vascular injury, as well as an inverse relation to vascular risk factors, but the exact mechanisms remained unclear. In addition, recent studies have implicated that DHEAS can lead to cell proliferation, angiogenesis, and protection against apoptosis. Therefore, the absence of DHEAS may cause inflammation and endothelial damage, contributing to cardiovascular disease [2,31]. Moreover, many have hypothesized that cardiovascular disease and DHEAS may share causal pathways, for example, nitric oxide synthesis and endothelial cell damage [31].

Contrarily, other studies have not found a direct correlation between them. For example, a community-based cohort study and a cross-sectional study in postmenopausal women did not find a relation between the DHEA levels and cardiovascular mortality, believing the metabolism of DHEA to testosterone may increase the risk for cardiovascular diseases [18]. As of date, the data are insufficient to predict whether DHEA is able to reduce the risk of cardiovascular diseases. Further research on DHEA and DHEAS involving cardiac pathologies and signaling pathways is necessary to clarify their associations with cardiac diseases [20,31].

### 4.3. Metabolism

Theoretically, DHEA and DHEAS have beneficial effects in regulating glucose and lipid metabolism and improving obesity. This kind of sex hormone is able to increase glucose clearance rate, oxidation of peroxidase activity, insulin production, and IGF-1 levels in plasma, and decrease tumor necrosis factor (TNF) production. Once DHEAS levels decline with age, there is an increasing prevalence of diabetes, which is why DHEA supplementation may be a new therapeutic way to restore impaired insulin signal transduction in skeletal muscle [16].

Nonetheless, several studies have reported no effect of oral DHEA therapy on insulin sensitivity due to inconsistent results [15]. DHEA circulates in the blood, mainly binding onto albumin and minimal binding onto sex hormone binding globulin [1]. In premenopausal women, inverse, positive, and nonsignificant correlations were respectively observed between DHEA, DHEAS concentrations, and body weight, body mass index (BMI), and ideal body weight percentage [32]. On the other hand, in postmenopausal women, the proposing androgenic effects of DHEA can lead to greater visceral fat accumulation due to hyperinsulinemia and decrease concentration in sex hormone binding globulin. Even low levels of DHEA can increase the incidence of metabolic syndrome [33]. Furthermore, in obese postmenopausal women, DHEAS has positive correlation with both fasting glucose and insulin resistance, as well as impaired glucose tolerance and diabetes [1,15]. An early report studied administration of 1600 mg DHEA for four weeks to obese postmenopausal women, and the results demonstrated that DHEA did not affect body fat mass, but the levels of total serum cholesterol and high-density lipoprotein cholesterol were significantly lowered. Furthermore, 1600 mg DHEA was able to induce an insulin-resistant status without acting on the fasting glucose levels due to its hyperandrogenic state [18]. In another study, daily oral administration of 50 mg DHEA for three weeks significantly reduced fasting triglycerides and enhanced insulin sensitivity, but did not affect body weight, body fat percentage, total cholesterol, low-density lipoprotein cholesterol, or high-density lipoprotein [1,18]. In an uncontrolled study, postmenopausal women applied 10% DHEA cream cutaneously daily for twelve months, and reported significant decrease in fat mass and increase in muscle mass [18]. For these study participants, conflicting conclusions may result from different research designs and DHEA dosage.

Lipid cell metabolism may play a role in frontal fibrosing alopecia. High levels of DHEA and DHEAS are found in women between the age of 25 and 30 years old, and decrease to 10% to 20% as women reach adrenopause, which is around the age of 60 years old. With the decrease in DHEA levels, developing frontal fibrosing alopecia has been present in adrenopause women. According to recent studies, DHEA is an essential stimulator for peroxisome proliferator-activated receptors (PPARs) in fat metabolism. Studies have revealed that the dysfunction of PPARγ, a main regulator of lipid cell metabolism, directly correlates to the fibrogenic inflammatory process of frontal fibrosing alopecia. The reduction of DHEA levels may be responsible for the follicular fibrogenic process. However, to date, there is no scientifically proven direct evidence on the role of DHEA in the connection between the fibrogenic process of fibrosis and the endocrine changes of adrenopause [34].

### 4.4. Immune System

Some functions of the immune system declines with age, which may be associated with the decrease in DHEA and IGF-1 levels. DHEA is capable of regulating interleukin synthesis and increase IGF-1 levels. Low levels of DHEA may be correlated with acquired immunodeficiency syndrome (AIDS) and immune disorders, such as systemic lupus erythematosus (SLE) and Sjögren’s syndrome [1,35].

Since neurosteroids are able to influence neurocognitive performance and modulate neuronal growth and survival, one study believes that enzymes controlling neurosteroid synthesis are suppressed in HIV infection, resulting in HIV related neurodegeneration. In HIV individuals, blood DHEAS levels are lowered. Proinflammatory genes, IL-1β, and TNF-α genes, contribute to neuronal injury and death, which ultimately lead to deficits in behavior and cognitive performance [12].

A number of studies reported the improvement in well-being, such as mood, fatigue, and energy in DHEA users, and believed the same may be applicable to aviremic patients with HIV, AIDS, and SLE patients [12]. In SLE patients, the secretion levels of type 1 cytokines, IL-2, and IFN are reduced, but the levels of type 2 cytokines, IL-6, IL-4, and IL-10 are increased. T cells in the peripheral blood are the major source of IL-2, IFN, and IL-4, whereas macrophages and monocytes are the primary source of IL-6 and IL-10. The imbalance is due to the defect in CD4+ Th1 cells or the overproduction of IL-6 and IL-10 [18]. SLE patients have low levels of DHEA, and the optimal dose of DHEA is unknown since there are inadequate studies on the immunologic effects of DHEA administered to humans [12]. However, with adequate doses of DHEA, modest improvements have been reported in several studies [3,19]. Casson et al. claimed that a daily oral dose of 50 mg DHEA for three weeks to postmenopausal women significantly increases the number and cytotoxicity of CD8+/CD56+ cells and a small decline in CD4+ T cells [18]. Treatment with DHEAS reduced virus expression, improved neurological performance, and lessened neurodegeneration and neuroinflammation [12].

Sjögren’s syndrome is an autoimmune disease and predominantly affects women at the age of 40 to 50 years. Symptoms of Sjögren’s syndrome include dry mouth, dry eyes, and infiltration of exocrine and epithelial tissues. During menopause, women experience a decline in estrogen levels; however, in women with Sjögren’s syndrome, the levels of estrogen are abnormally high. DHEA can be found in healthy salivary gland epithelial cells, but in women with Sjögren’s syndrome, the levels of DHEAS are low. Along with this dysregulation, the findings of low DHEAS levels may also support the reason why local androgen deficiency can be seen in salivary glands. Low levels of 3β- and 17β-hydroxysteroid dehydrogenases are also reported since they lose their basal acinar cell localization. Women with Sjögren’s syndrome may feel vulnerable due to the dysfunction since local DHT production is dependent on DHEA, which explains the low local DHT and androgen biomarker levels [36].

Despite the fact that several patients have been taking DHEA as dietary supplements, these steroid hormones have not undergone rigorous testing for their efficacy as therapeutic agents. There is evidence suggesting DHEA may have a role in the treatment of autoimmune diseases, but there is no evidence to support potential efficacy of DHEA supplementation as a treatment for Sjögren’s syndrome. Adverse effects from prolonged usage of unregulated DHEA supplements are unknown; therefore, DHEA should not be prescribed [35].

### 4.5. Bone

DHEA can be expressed via sex steroid receptors, androgen and estrogen receptors, and are present in the mRNA pathway of growth plate tissues [37]. The activation of androgen receptors can stimulate bone cell proliferation and differentiation [15]. The role of DHEA in bone mineral density can be mediated by increasing testosterone and androgenic effects.

On the other hand, recent studies have indicated DHEA plays a role in growth plate homeostasis by suppressing longitudinal bone growth through estrogen receptors, inhibiting chondrocyte proliferation and differentiation, and inducing chondrocyte apoptosis. In order to prevent persistent cell apoptosis, DHEA is able to transmit its signal via Gαi-PI3K/Akt-Bcl-2 pathway. The suppression effect is mediated through the estrogen receptors, especially ERβ, via NF-kB signaling pathway. Since the estrogen receptor and NF-kB signaling pathways are associated with one another, activation of the estrogen receptor can inhibit both NF-kB activation and gene expression. Estrogen receptors, ERα and ERβ, are expressed in growth plate tissues, meaning estrogens can directly act on growth plate chondrocytes. ERα mediates growth-promoting effects of estradiol in pubertal bone, but does not involve in maintenance of trabecular bone. Contrarily, ERβ terminates growth spurts in females during puberty, which limits longitudinal and radial bone growth [37]. Not only can 17β-estradiol-3-benzoate (E2) activate the pathway in bone and be involved in bone turnover, DHEA is able to activate the classical estrogen-signaling pathway in bone and thymus, which suggests DHEA may act the same way on trabecular bone mineral density [9].

Many suggest there is a protective role for DHEA against osteoporosis, because the decline in DHEA and DHEAS levels with aging is parallel to the decrease in bone mineral density [1,18]. However, several studies have found this hypothesis quite contradictory. In two population-based studies on postmenopausal women, there is a positive relation between DHEAS and spinal bone mass, but in postmenopausal women with rheumatoid arthritis, there is one study with positive correlation and another with negative correlation between DHEAS levels and bone density [18]. According to these four studies evaluating the effects of DHEA on spinal bone mineral density in postmenopausal women, there is no definitely beneficial effect, and this conclusion could possibly be due to the potential risks of bias and imprecision. Moreover, the effects of DHEA on fracture prevention in postmenopausal women have not been fully evaluated. Thus, further studies must be conducted before DHEA can be considered a therapeutic option for the management of bone mineral density.

### 4.6. Cancer

Studies report that postmenopausal women with higher concentrations of DHEA are at higher risk of breast cancer, whereas premenopausal women with low DHEA concentrations are at risk.

The proposal behind this correlation is that in premenopausal women, DHEA has anti-estrogenic properties, which inhibits tumor cell growth [32]. In postmenopausal women, DHEA can serve as an estrogen agonist with an ability to stimulate breast cancer cells [1,32]. The estrogen concentration in breast tumors is significantly higher compared to the plasma levels, which could be from the conversion of androgens to estrogens by the aromatase enzyme (CYP19) [38]. Based on these findings, some even suggest that breast cancer in postmenopausal women is stimulated by prolonged intake of DHEA [39].

DHEA has both inhibitive and proliferative effects on breast cancer cells, depending on the patient’s age, the intimate estrogen status, the dosage, and the distribution of estrogen or androgen receptors. The inhibition of androgen and estrogen receptors on human breast cancer MCF-7 cells does not change the inhibitive and proliferative effects of DHEA. DHEA is able to inhibit cell proliferation by itself, which cannot be replaced with testosterone or 17β-estradiol [39]. 5-androstene-3β,17β-diol (5-diol) is structurally an androgen, but binds to both androgen receptor and estrogen receptor, and appears as a weak estrogen. Improvements has been shown in breast cancer patients who are treated with sulfatase inhibitors because of inhibiting the conversion from DHEAS to DHEA, and leading to insignificant levels of 5-diol [15]. Although DHEA is an androgen with potentially protective effects, and has the ability to diminish cell proliferation; however, its exact mechanism of action remains unclear. Due to this evaluation, DHEA at therapeutic doses could possibly be used in the treatment of breast cancer.

### 4.7. Cognition

In terms of cognitive effects, both DHEA and DHEAS may enhance the sense of well-being. Both concentrations are high in the brain and can be synthesized from cholesterol. DHEA is able to penetrate the blood-brain barrier and acts as an inhibitor of GABA receptors [1]. The exact mechanism underlying the effect of DHEA in the central nervous system is still under investigation, but low levels of DHEAS correlate with the degree of dependence in daily activities, and are seen in people with poor health due to stress-related immunological dysregulation [22].

Alzheimer’s disease is a progressive neurodegenerative disorder, which affects memory, behavior, and personality. Calcium-phospholipid-dependent protein kinase C (PKC) alters the learning and memory mechanism, and defective PKC translocation is associated with age-dependent cognitive impairment. Individuals who suffer from Alzheimer’s disease have diminishing levels of RACK-1 protein and decreasing concentrations in plasma DHEA and DHEAS [5,6]. In addition, one study claimed increased DHEA concentrations are found in Alzheimer’s individuals as a form of protection by means of the oxidative stress-mediated metabolism [6]. Prolonged cortisol elevations and higher basal cortisol concentrations can cause hippocampal damage, while DHEA can have anti-glucocorticoid effects. Hence, Alzheimer’s disease patients can take DHEA medication to protect the hippocampus [40]. The efficacy of treating the symptoms with DHEA is still unknown, but DHEA can enhance hippocampal cholinergic cell function, and protect against oxidative stress by exerting anti-glucocorticoid effects, increasing IGF-I, and decreasing production of amyloid β protein [41].

The relationship between DHEAS and depression remains to be proved. One study assessed different concentrations of steroid hormones and revealed that only DHEAS concentrations were inversely correlated with depression, and were independent from other factors such as age, body weight, exercise, and gender. Women who experienced first onset of depression during perimenopause showed both low morning DHEA and DHEAS concentrations [21]. Significant improvement in depression scale ratings has been reported in depressed individuals undergoing DHEA treatment, suggesting that DHEA may have antidepressant effects [5]. Nonetheless, stress and glucocorticoids also play important roles in memory and cognitive performances in depression, posttraumatic stress disorder, and dementia. Therefore, further studies with large-scale clinical trials will be needed to ensure DHEA’s efficacy [40]. However, there is no direct evidence that DHEA therapy can benefit cognitive performance or prevent cognitive decline in people over the age of fifty [5,15]. Moreover, gathered data do not specify that DHEA is a cognitive enhancer in healthy seniors, possibly due to inadequate sample sizes and variant research designs [5]. To investigate the cognitive effects of DHEA and DHEAS on healthy individuals, large-scale studies are warranted.

### 4.8. Infertility, In Vitro Fertilization (IVF), and Polycystic Ovary Syndrome (PCOS)

Naturally, DHEA concentrations peak in the mid-20s, but then progressively decline. Once the DHEA level is low, it is unable to enhance replication and transcription of DNA, and the reproductive tissues are detrimentally affected in the process of fertilization and conception. Furthermore, DHEA has the ability to protect against oxidative stress. Due to changes in local perfusion and remodeling during pregnancy, high levels of reactive oxygen species (ROS) are produced in the uterus, thus resulting in sensitivity and hypoxia to the preimplantation embryo, and causing detrimental effects to the development of the embryo. In order to protect against oxidative stress, low concentration of DHEA can inhibit further ROS production, enhance endometrial receptivity, and improve embryo implantation [42]. Moreover, DHEA is able to increase FSH receptor expression in granulosa cells, increase IGF-1 level, promote primordial follicular growth, and increase pre-antral and small antral follicles [43,44].

The postponement of childbearing has led to subfertility and it is related to a diminished ovarian reserve. As an indicator of ovarian aging, diminished ovarian reserve correlates with reductions in quantity and quality of oocytes, and this becomes prominent after the age of 38 [44,45]. As the ovarian reserve diminishes, inhibin B secreted by granulosa cells decreases, and FSH concentration increases and stimulates follicle development. Elevated FSH in different reproductive ages has different response and likelihood of pregnancy; compared to women of advanced age, younger women with elevated serum FSH and accelerated follicle growth will have more live birth pregnancies [44].

DHEA is capable of improving the ovarian response and the quantity and quality of oocytes; therefore, it is used to treat infertile patients undergoing in vitro fertilization (IVF). In one study, homeobox A10 (HOXA10), a homeodomain transcription factor in the endometrium for embryo implantation, is regulated by estrogen and progesterone, and is speculated to be regulated by DHEA via androgen receptor [42]. Due to the important roles in female reproductive physiology and in follicular steroid biosynthesis, androgens have been proposed as an adjunctive treatment in poor responders [46]. Androgen metabolization and concentrations matter most in women with premature ovarian aging. Metabolizing DHEA into testosterone significantly improves the chances of becoming pregnant. On the other hand, the use of androgen supplementations in poor responders remains controversial since hyperandrogenism, excessive follicle count, and characteristic features of polycystic ovary syndrome (PCOS) can cause adverse effects on follicle maturation [47]. Individuals with PCOS have abnormal levels of growth factor, high levels of luteinizing hormone (LH) and androgen excess from ovaries and adrenal glands. According to folliculogenesis, LH is also essential in the final stage of follicular maturation. By increasing the intrafollicular androgen concentration in the early follicular phase, there will be an increase in good embryos [48]. Throughout the years, optimal levels of DHEA supplementation have been searched to improve the outcomes of pregnancy for women with diminishing ovarian reserve. Several studies have reported an increase in ovarian responses and improved pregnancy outcomes after DHEA supplementation. Speculations have been made that DHEA might affect ovarian follicular growth by acting as a ligand for androgen receptors and as a metabolic precursor for steroid production [45,48]. For example, two clinical cases of poor responder patients with prematurely developing antral follicles have successful pregnancies based on follicle sizes [44]. Treatments with DHEA have been proposed for improving ovarian reserve, restoring androgenic ovarian microenvironments, and improving the outcome of IVF pregnancy rates, such as an increase in retrieved oocytes, clinical pregnancy rate, and live birth rate. However, there is lack of absolute evidence supporting its effectiveness [42,43,45,46,47].

Moreover, the manifestations in hyperandrogenism include high levels of testosterone, δ-4 androstenedione, DHEA, and DHEAS. DHEA levels in PCOS patients have abnormal correlation with abdominal obesity, dyslipidemia, and insulin resistance. In other words, women with PCOS commonly have insulin-resistance with hyperinsulinemia, as well as increased risk of impaired glucose tolerance and type 2 diabetes mellitus. However, a population-based, prospective cohort study showed that reduced levels of serum DHEA increase the risk of type 2 diabetes, due to the possible mechanism in increasing glucose uptake after the stimulation of GLUT4 and GLUT1 translocation to the plasma membrane [49]. As mentioned above, DHEA has the ability to inhibit oxidative stress, activate PKC, and activate phosphatidylinositol 3-kinase (PI3-kinase) to improve glucose uptake and improve endothelial function. In addition, statins are able to reduce cholesterol synthesis, inhibit synthesis of adrenal and ovarian androgens, and inhibit insulin secretion. Nevertheless, data focusing on the mechanism and effects of statins on DHEA levels in PCOS patients have been inconsistent. For example, a meta-analysis by Yang et al. showed that statins might reduce the levels of DHEA, reduce insulin sensitivity, and increase the risk of diabetes, while other studies claimed that statins improved insulin resistance and increased insulin sensitivity in patients with PCOS [49]. More investigations are needed to draw a reliable conclusion.

### 4.9. Adrenal Insufficiency

Individuals with adrenal insufficiency show impaired quality of life, higher levels of depression, and reduced birth rates and ovarian autoimmunity [3]. A lack of DHEA secretion leads to a downfall in circulating DHEA and DHEAS levels, which can be found in women with significant androgen deficiency. Nonetheless, in postmenopausal women with normal adrenal functions, DHEA usage has no association with significant improvement in sexual function, quality of life, and general well-being [29].

Deficiencies of glucocorticoid and mineralocorticoid in primary adrenal insufficiency require lifelong replacement, but the use of DHEA replacement therapy is still equivocal due to insufficient and different results on quality of life and well-being. Some studies have reported positive effects of DHEA replacement therapy in women with adrenal insufficiency, such as remarkable improvement in mood, depression, and sexual function, as well as increase in serum androgen [50]; however, the use of DHEA replacement is not the standard treatment while considering its side effects, like skin fat content and hyperandrogenism [3]. Studies have suggested that well-being of women with primary adrenal insufficiency is caused by DHEA-increasing IGF-1 production, which is dependent on growth hormone, and have shown that individuals have benefited from an oral dose of 50 mg DHEA per day [50]. Arlt et al. conducted a randomized, placebo-controlled, double-blind crossover trial over a period of four months and reported 14 out of 24 women with adrenal insufficiency significantly improved in well-being and sexuality after taking DHEA [3,19]. Another randomized placebo-controlled double-blinded study conducted by Hunt et al. reported improvement in mood and fatigue after using DHEA. On the contrary, the randomized placebo-controlled trial of 39 women by Lovas et al. did not obtain beneficial effects on health status or sexuality [3]. Similarly, Gurnell et al. also reported no beneficial effects of DHEA treatment on fatigue and sexual function, but positive effects on bone mineral density and body composition during a 12-month trial [3,19]. Although there is evidence from some randomized, placebo-controlled clinical trials that DHEA replacement therapy has shown significant benefits on mood, sexuality, and quality of life in women with adrenal insufficiency, further studies with prolonged observation periods and dosage adjustments are desirable [3,51]. Currently, there is no complete evidence to support the use of DHEA replacement therapy in women with adrenal failure since the existing studies are scant and ambiguous [8].

### 4.10. Weight Training on Muscle Mass and Strength

Deficiencies of steroid hormones can hinder skeletal muscle development. With age-related hormone decrease, a progressive decline in muscle mass and strength can occur. DHEA replacement can cause small increases in testosterone and IGF-1 levels, which assists muscle strength and mass [10]. In addition, DHEA replacement can decrease intra-abdominal fat, increase insulin action and glucose tolerance, and increase muscle mass and strength caused by resistance training. According to another study, there is a threefold increase in testosterone levels, but the absolute level of testosterone in women is still very low [7]. Further studies should investigate the gene expression of steroid sulfatase in skeletal muscles and aromatase cytochrome P-450 due to the crucial role it plays in sex steroid metabolism [10].

### 4.11. Effects on General Well-Being

Multiple studies have found lowered serum concentrations of DHEAS in patients with poor life quality, psychosocial stress, and functional impairment. Higher concentrations of DHEAS have been connected to better functioning, greater enjoyment of leisure activities, and overall higher life satisfaction. Blood DHEAS concentrations are believed to have positive correlation to the liveliness of premenopausal women, but are unrelated in postmenopausal women. Nonetheless, in another study, 80% of postmenopausal women undergoing DHEA treatment reported improved well-being and vitality [21].

In the adrenal gland, DHEA is produced from cholesterol through a pregnenolone pathway and converted to testosterone and estrogen; on the other hand, glucocorticoids are metabolized during stress. Chronic fatigue syndrome is an illness that leads to depression, changes in mental-emotional capacity, and decrease in daily activity. Abnormal production of adrenal hormone cortisol and DHEA were reported in individuals with chronic fatigue syndrome, thus suboptimal production of DHEA might be prevalent in these individuals. By taking DHEA supplements, these patients may significantly improve their overall well-being [4].

In a certain way, cortisol can serve as the antagonist of DHEA and prepare for the fight or flight mechanism. Interestingly, ginseng has the ability to reduce the production of cortisol and the cortisol to DHEA ratio, thus enhancing the relative effectiveness of DHEA [52].

### 4.12. Major Depressive Disorders

In comparison with men, women are more likely to be diagnosed with depression. Previous research revealed that sadness, guilt, and discouragement are correlated with the decrease of serum DHEA levels. The mechanism underlying this observation is possibly that DHEA decreases plasma cortical levels and exerts antidepressant effects via its interaction with noradrenaline and serotonergic neural systems and σ-1 receptors [53]. Both DHEA and DHEAS play an important role in affecting the central nervous system and impacting mood, stress levels, and mental health, but they should not be definite indicators for assessing the severity of depression.

### 4.13. Stress

Major life stressors include time-dependent, type-dependent, and cumulative stress exposure. Time-dependent life stressors classified into acute life events and chronic difficulties, are different from type-dependent stressors, which include recent life stress and childhood-specific stressors. Studies have found that people are prone to stress-related health problems when they experience more major life stressors. For example, particular life events may cause the onset of depression, which may be associated with worse cognitive function, or may even speed up biological aging. Cumulative stress exposure is the accumulation of acute life events and chronic difficulties during one’s lifespan; however, this form of stress is difficult to evaluate since there has not been any study exploring the assessment in relation to biological responses to stress. Recently, the Stress and Adversity Inventory for Adults (Adult STRAIN), a predictor of stress-related outcomes, has been an efficient instrument for assessing cumulative stress exposure and related outcomes, such as poor mental and physical health, diseases, disorders, and cognitive functions [54].

When one is stressed, the adrenal glands in one’s body upregulate and release cortisol and DHEA. These hormones play a part in glucose metabolism and in the immune system, and help regulate each other. Without the function of regulating each other, inflammation and many physical and mental health problems may occur. DHEA can exert both anti-glucocorticoid and anti-inflammatory effects in response to acute stress, and has a positive correlation with cognitive function. Likewise, when there are lower levels of DHEA, one is more likely to be depressed or subject to psychiatric disorder. Lam et al. examined the associations of cumulative stress exposure with cortisol and DHEA, and found more exposure to cumulative stress foresaw blunted cortisol levels and enhanced DHEA responses to acute stress. This study is the first one to research cumulative life stress exposure and the association between cortisol and DHEA responses to stressors of different types and timing of the exposure. Exposure to the stressors predicted cortisol and DHEA responses to acute stress, while only adulthood stressors predicted cortisol response to acute stress. Although both levels of DHEA and cortisol increase following ACTH stimulation, this association can be explained by the regulation of each other. DHEA increases to suppress cortisol level in response to stress, which is why those with depression, schizophrenia, and post-traumatic stress disorder have abnormal cortisol to DHEA ratio. The correlation among cortisol, acute stress, and DHEA in terms of the effects on lifetime stress is currently under-researched, but may play an important role in shaping health and disease [54].

### 4.14. Fasting-Induced Hepatic Production

Studies have shown DHEA can lower abdominal visceral fat, insulin levels, and the concentration of free amino acids in hepatic cells. During fasting, DHEA also protects hepatic cells from elevated oxidative states. PGC-1α is a key integrator that regulates steroid production in the liver and induces gene expressions of CYP11A1 and CYP17A1. By inducing hepatic metabolism, both of these aforementioned gene expressions convert cholesterol into pregnenolone and then into DHEA. CYP11A1 is able to generate cholesterol metabolites and regulate homeostasis and lipid metabolism. CYP17A1 is able to produce squalene epoxide. Both enzymes can produce liver X receptor (LXR) ligands, potentially providing a mechanism by which fasting and PGC-1α can regulate LXR activity. Although the specific receptor of DHEA has not been recognized, DHEA protects endothelial cells from apoptosis via Gαi receptor-mediated induction of PI 3-kinase/Akt-mediated pathway, and the Akt pathway in hepatic cells inhibits PGC-1α activity [38].

## 5. Discussion

As women age, they experience a drop in DHEA levels, which are potentially linked to hormone deficiency, postmenopausal symptoms, and many age-related diseases. There is a possibility that those who are healthier are more prone to have higher DHEA levels. DHEA has important functions in the human body, but it is crucial to ask oneself whether being healthy or having nourishing DHEA levels is a priority. Many may get a hold of DHEA supplements in hopes of improving libido and sense of well-being. Since DHEA is able to convert to androgen or estrogen, many propose DHEA replacement therapy may yield beneficial effects, such as improving menopausal vasomotor symptoms when converted to estrogen, increasing libido and boosting well-being from its androgenic effects. With a daily dose of 50 mg of oral DHEA, data suggest DHEA supplements may have a role in preserving the integrity of the immune system by attacking cancer cells and viruses, maximizing anti-cancer function, and enhancing the activity of monocytes, especially in individuals with autoimmune diseases. Moreover, due to inhibitory effect on the development of mammary carcinoma, DHEA may be used to treat breast cancer. However, further investigation is needed [15]. Furthermore, administration of DHEA may increase muscle and strength and be used to treat infertile patients by improving ovarian response and improving the quantity and quality of oocytes. Unlike oral DHEA, intravaginal administration of 0.50% DHEA has shown significant beneficial effects in postmenopausal women with severe vulvovaginal symptoms.

On the other hand, several randomized controlled trials have reported conflicting results on the effects of DHEA. A number of reviews assessing the benefits and harm of administering DHEA in postmenopausal women with normal adrenal function have reported insignificant improvement in libido, sexual function, well-being, cognitive performance, serum lipids, glucose, and bone mineral density. Moreover, qualitative summary states that oral DHEA therapy does not support benefits for postmenopausal women because of the results warranting low confidence [29]. In terms of cardiovascular diseases and adrenal insufficiency, no RCT with sufficient size has shown consistent and safe evidence of oral DHEA in women. Likewise, most of the studies reported no effect of oral DHEA on lipid profiles and insulin sensitivity. Moreover, oral DHEA has shown modest effects on bone density in individuals with osteoporosis, but large-scale studies on fracture prevention have not been conducted. The effect of DHEA on cognition is not as promising since well-designed studies have not totally proven its ability to enhance cognitive performance. In addition, administering DHEA to depressed patients has positive effects, but it should be made with caution due to limited evidence with small sample sizes. It is also noted that those with schizophrenia and post-traumatic stress disorders may benefit from DHEA, but the exact role of DHEA in acute stress remains unclear. In contrast, Fedorkow claims that DHEA does not improve quality of life, menopausal symptoms, and sexual function in peri- and postmenopausal women, but results in increased androgenic side effects, such as acne and hirsutism, which could perhaps do more harm than good [55].

It is not practical to normalize DHEA and DHEAS concentrations in healthy individuals as a way to slow down or prevent the advancement of age-related diseases. Favorable effects from DHEA treatment are more apparent in medically ill patients compared to healthy individuals. In recent years, there has been an ongoing rise in DHEA research; however, DHEA is still not well understood due to inconsistent clinical findings of its mechanisms of action, roles in different diseases, and the optimal dosage. Limited effectiveness of DHEA therapy in postmenopausal women may origin from an insufficient amount of enzymes required to convert DHEA to estrogens and androgens. Anyhow, large-scale randomized, controlled trials are necessary for a better understanding.

In general, postmenopausal women do not benefit from oral DHEA, and women with intact adrenal function do not necessarily benefit from DHEA supplementation. Some clinical trials have suggested that DHEA treatment increases cognitive performance, sense of well-being or mood, and sexual function in both healthy elderly and those with illnesses, whereas other studies have shown no therapeutic effect. Currently, there are no definitive conclusions regarding the effects of DHEA on humans due to limited evidence. Studies on DHEA have not been tested over a long interval of time, so long-term effects of DHEA are unknown. Side effects of DHEA usage may vary according to gender, age groups, and diseases. Anyone taking DHEA supplements should take precautions since there is a lack of direct evidence of effectiveness and safety.

To minimize the heterogeneity of DHEA studies, standardization of several associated factors should be considered in the future. One of the associated factors is “time to menopause”, which has a major impact on the individual response to DHEA. Among future studies, the DHEA dosage needs to maintain consistency for a superior comparison of the therapeutical effects, and for ensuring the reliability. Furthermore, the study duration and outcome measure also need standardization, along with important endocrinal profiles of enrolled women. Moreover, larger sample sizes are required to draw reliable conclusions and improve the validity and replicability of the study results.

Due to either religious practices or long-standing customs, traditional diets in many Oriental countries include vegetables and more vegetables, strong spices, rice and noodles, seafood, and soy products. On the other hand, the Mediterranean diet is usually low in the intake of meat, and high in vegetables, fruits, legumes, nuts, beans, cereals, grains, fish, and unsaturated fats such as olive oil. Both Oriental and Mediterranean diets have been associated with good health, and possibly alleviating the menopausal syndromes, because of the isoflavone contents in the soybeans. As one of the nutraceutics, isoflavone has the active ingredients including daidzein, genistein, and S-equol, and can reduce vasomotor syndromes such as hot flashes and sweating. Although the aforementioned diets and physical activity cannot increase the synthesis of DHEA, their combinations seem effective at ameliorating the vasomotor symptoms, and they are considered as part of a future direction toward counteracting menopausal syndromes in affected women.

## 6. Conclusions

By focusing on anti-aging properties of DHEA, it may be appealing to take DHEA during midlife or late life since this hormone is sold over the counter and on the internet. However, DHEA supplements for both premenopausal and postmenopausal women are not advised due to unknown long-term effects. DHEA is a precursor for many androgenic and estrogenic entities, and its metabolic pathways may differ between genders and among ages. The efficacy of DHEA has been studied in inadequate sample sizes, thus yielding inconsistent results in postmenopausal women. Therefore, large-scale randomized, controlled trials, with comparable dosages and adequate treatment durations are warranted to further understand the risks and benefits of DHEA, and draw reliable conclusions. Recent randomized controlled trials on the use of vaginal DHEA in women have shown beneficial results in vaginal atrophy and possible improvement in sexual function; however, these trials do not support the benefits of oral DHEA therapy. Since no benefits are demonstrated at this time, and larger sample sizes are necessary to assess the safety and efficacy of DHEA, oral DHEA therapy is not mandatory in premenopausal and postmenopausal women, but may provide as a therapeutic option in the future.

## Data Availability

Data is contained within the article.

## Abbreviations

P450	Cytochrome P450
CYP17A1	Cytochrome P450 17A1
17α-OHPreg	17α-hydroxypregnenolone
17α-OHProg	17α-hydroxyprogesterone
DHEA	Dehydroepiandrosterone
DHEAS	Dehydroepiandrosterone sulfate
3β-HSD	3-beta (β)-hydroxysteroid dehydrogenase
17β-HSD	17-beta (β)-hydroxysteroid dehydrogenase
DHT	Dihydrotestosterone
3α-diol	3α-androstanediol
3α-diol-3G	androstane-3α,l7β-diol-3-glucuronide
PAPS	3’-Phosphoadenosine-5’-phosphosulfate
SULT2A1	Sulfotransferase Family 2A Member 1
GABA	γ-aminobutyric acid
$\sigma_{1}$ receptor	Sigma-1 receptor
NMDA	N-Methyl-D-aspartic acid or N-Methyl-D-aspartate
Akt kinase	Protein kinase B (PKB)
